# Clinical map document based on XML (cMDX): document architecture with mapping feature for reporting and analysing prostate cancer in radical prostatectomy specimens

**DOI:** 10.1186/1472-6947-10-71

**Published:** 2010-11-15

**Authors:** Okyaz Eminaga, Reemt Hinkelammert, Axel Semjonow, Joerg Neumann, Mahmoud Abbas, Thomas Koepke, Olaf Bettendorf, Elke Eltze, Martin Dugas

**Affiliations:** 1 Prostate Center, Dept. of Urology, University Hospital Muenster, Albert-Schweitzer-Str. 33, D-48149 Muenster, Germany; 2 Prostate Center, Gerhard-Domagk Institute for Pathology, University Hospital Muenster, Domagkstr. 17, D-48149 Muenster, Germany; 3 Institute of Pathology and Cytology, Technikerstrasse 14, D-48465 Schüttorf, Germany; 4 Institute for Pathology, Saarbrücken-Rastpfuhl, Rheinstrasse 2, D-66113 Saarbrücken, Germany; 5 Department of Medical Informatics and Biomathematics, University of Muenster, Domagkstr. 9, D-48149 Muenster, Germany

## Abstract

**Background:**

The pathology report of radical prostatectomy specimens plays an important role in clinical decisions and the prognostic evaluation in Prostate Cancer (PCa). The anatomical schema is a helpful tool to document PCa extension for clinical and research purposes. To achieve electronic documentation and analysis, an appropriate documentation model for anatomical schemas is needed. For this purpose we developed cMDX.

**Methods:**

The document architecture of cMDX was designed according to Open Packaging Conventions by separating the whole data into template data and patient data. Analogue custom XML elements were considered to harmonize the graphical representation (e.g. tumour extension) with the textual data (e.g. histological patterns). The graphical documentation was based on the four-layer visualization model that forms the interaction between different custom XML elements. Sensible personal data were encrypted with a 256-bit cryptographic algorithm to avoid misuse. In order to assess the clinical value, we retrospectively analysed the tumour extension in 255 patients after radical prostatectomy.

**Results:**

The pathology report with cMDX can represent pathological findings of the prostate in schematic styles. Such reports can be integrated into the hospital information system. "cMDX" documents can be converted into different data formats like text, graphics and PDF. Supplementary tools like cMDX Editor and an analyser tool were implemented. The graphical analysis of 255 prostatectomy specimens showed that PCa were mostly localized in the peripheral zone (Mean: 73% ± 25). 54% of PCa showed a multifocal growth pattern.

**Conclusions:**

cMDX can be used for routine histopathological reporting of radical prostatectomy specimens and provide data for scientific analysis.

## Background

**Figure 1:**
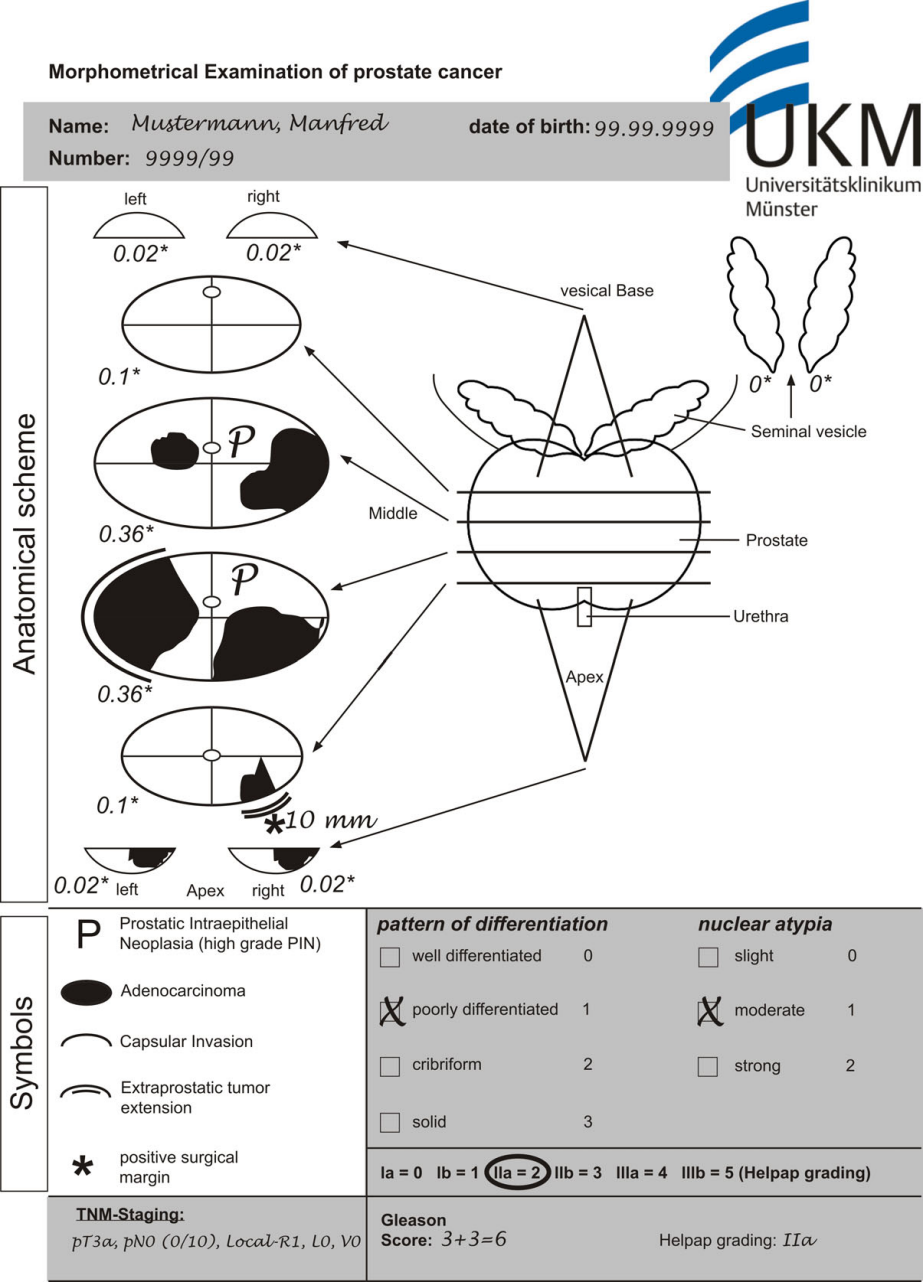
**Pathology report of a radical prostatectomy specimen in paper form**. The prostate is sliced by a standard method. The histopathological findings in a slice are depicted in the corresponding slice of the scheme. Each slice is attributed its own "slice factor" (^+^) representing the proportion. It is contributing to the whole prostate volume. There are two types of information: graphical (white background) and textual (grey background). The graphical information includes the schematic diagram and the symbols, whereas personal and pathological data is textual.

Prostate Cancer (PCa) is the most commonly diagnosed cancer in men and one of the leading causes of cancer deaths in Germany [1]. As therapeutic approach, many patients choose total removal of the prostatic gland (radical prostatectomy). Pathology reports of radical prostatectomy specimens include clinically relevant information as well as clinically essential information derived from the macroscopic examination and microscopic evaluation, which play a supporting role in clinical decision making and prognostic evaluation of PCa [2,3]. Consequently, diverse standardized sectioning and documentation protocols of radical prostatectomy specimens are described [4-7]. In our center, we use the standardized pathologic report according to Bettendorf (Figure 1) [6]. This report includes a diagrammatic representation of histopathological findings in the prostate gland. It is a practicable method which documents tumour extension, extracapsular tumour growth, and the status of surgical margins in radical prostatectomy specimens.

To our knowledge, there is no established electronic standard for graphical documentation of PCa that meets clinical and research requirements. These requirements include a flexible documentation of PCa extension with an anatomical schema that can be used for clinical and research purposes. To provide an analyzable data acquisition model for anatomical schemas in electronic form, we propose a document architecture called "clinical Map Document based on XML" (cMDX). The development of this document architecture depends on clear definitions of domain terminologies, functional and data hierarchies, as well as assessment rules. It is intended to improve information consistency and data integrity of routine data in hospital information systems and clinical studies.

This article describes a data model based on schematic diagrams for documentation and analysis of prostatectomy specimen reports.

## Methods

**Figure 2:**
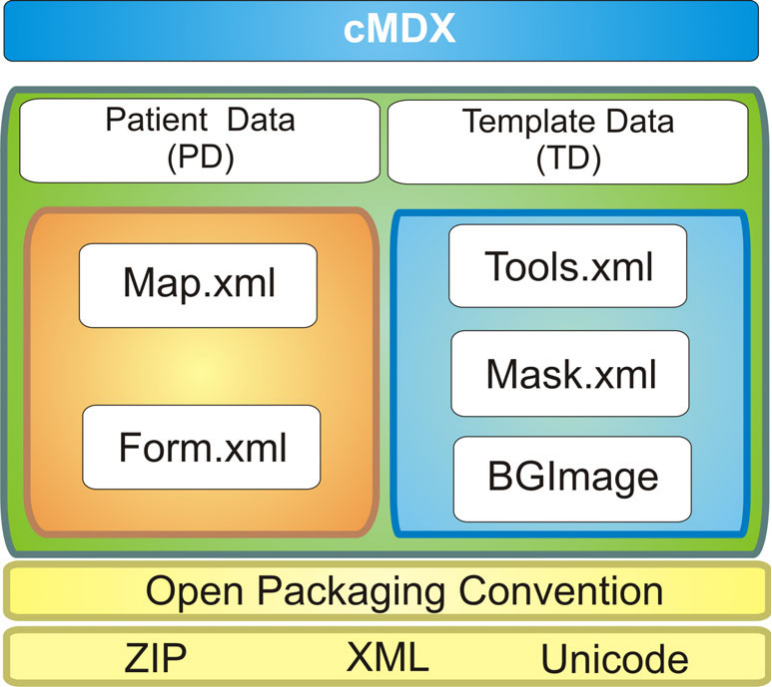
**The components of the cMDX document architecture**.

**Figure 3:**
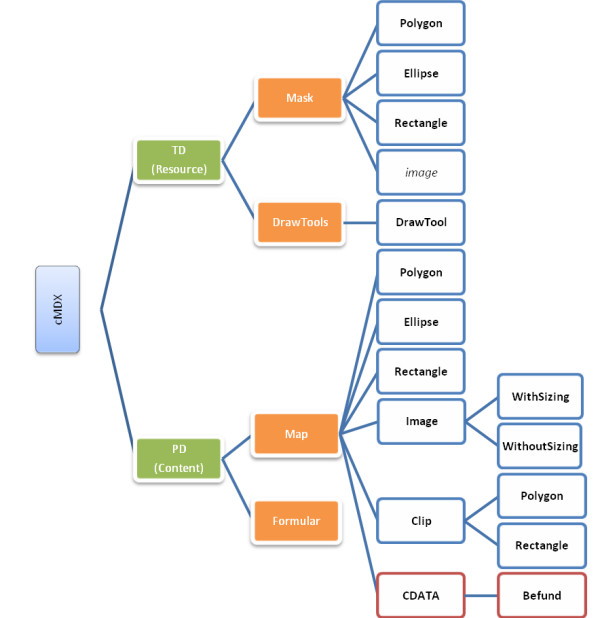
**Simplified tree diagram of cMDX elements**. TD: Template Data; PD: Patient Data; The folder "Resource" in the zip package of cMDX stores the document part TD, and the folder "Content" the document part PD. The CDATA section in a Map sub element contains a "**Befund**" element describing the histological patterns of PCa.

**Figure 4:**
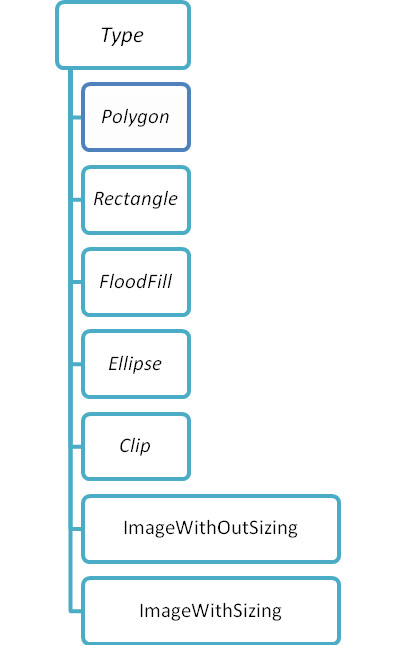
**The attributes and values of the DrawTool element**. If, for instance, the value of the attribute *Type *equals "Polygon," a PCa with polygonic shape can be represented.

**Figure 5:**
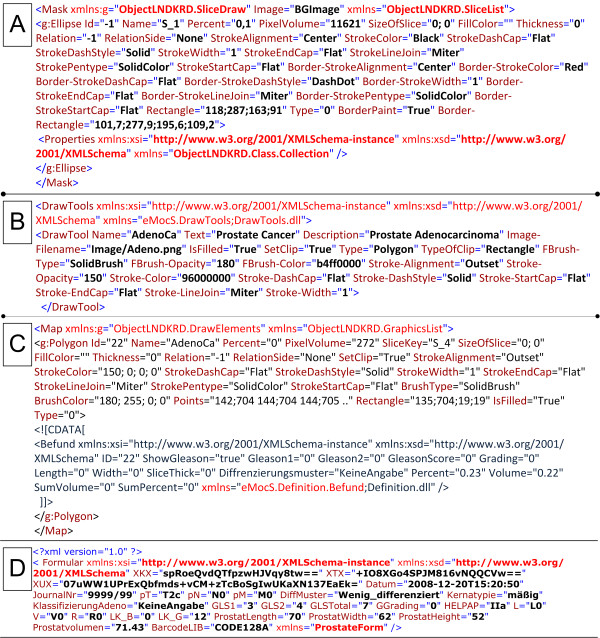
**Examples of the syntax of cMDX elements**. A: Mask. B: Tools. C: Map. D: Form with encrypted patient data.

**Figure 6:**
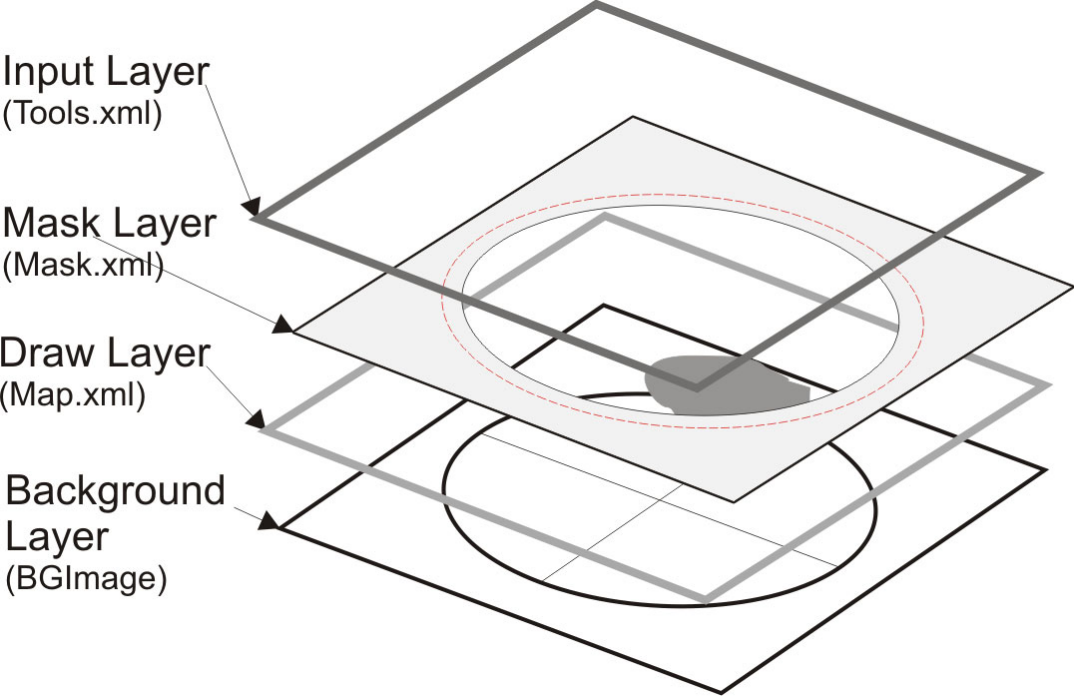
**Visualization Model: Four-Layer Model**. The workflow of the Four-Layer model: (1) The Input Layer registers the cursor movement and the drawing tool selected by the user, thereby calling functions for user guidance or data acquisition. (2) The Mask Layer defines the drawing surface. (3) The Drawing Layer represents the drawing elements resulting from layers 1 and 2. (4) The Background Layer contains schematic diagrams with graphical descriptions. The red lines in the Mask Layer are defined by attributes beginning with the word "Border" (see table 1). These red lines show the boundary of a drawing surface on which shapes and symbols can be drawn.

To develop a data acquisition model for the histopathological examination report, the requirements for the documentation system were collected by unstructured interviews with three urologists and three pathologists. Additionally, twenty reports in paper form were analysed. Thereafter, a multiple-layer model based on XML (Extensible Markup Language) specifications and vector graphics was designed in order to generate similar reports in electronic form. Figures 2, 3, 4, 5 and 6 illustrate the data structure that is explained in detail in the following sections. Table 1 describes the presentation attributes; Tables 2 and 3 show attributes and textual information included in this model. In this paper, terms written in **bold **or *italic *represent either XML **element **or *attribute *names. Element and attribute names are concatenated with the first letter of each word capitalised (UpperCamelCase) or with a dash (String-UpperCamelCase). Supplementary tools for cMDX were written in C# with Microsoft^© ^Visual Studio 2008. The electronic Hospital Information System (HIS) Orbis™used in the University Hospital Muenster is provided by AFGA Health Care^©^. All patient records are currently stepwise transformed from paper documentation to entirely electronic documentation.

**Table 1:** List of presentation attributes.

Attributes/ Elements	Description	Value Type	Example
*IsFilled*	If true, then fill the shape otherwise not.	Boolean	True
*SetClip*	If true, show only the intersected drawing surface with mask.	Boolean	True
*BrushType*	Specifies the type of the brush (Hatchbrush, Solidbrush)	String	SolidBrush
BrushOpacity*	Set the colour opacity of filled area.	Integer	180
*BrushColor*	Set the fill colour (Alpha;Red;Blue;Green)	Colour	180; 255; 0; 0
*Stroke-Alignment*	Specifies the alignment of lines in relation to the theoretical, zero-width line	String	Outset
StrokeOpacity*	Setting the opacity of lines (0-255)	Integer	150
*StrokeColor*	Setting the colour of lines (A, R, B, G)	Colour	150; 0; 0; 0
*StrokeDashstyle*	Specifies the style of dashed lines	String	Flat
*StrokeStartCap*	Specifies the available cap styles of the beginning of a line.	String	Solid
*StrokeEndCap*	Specifies the available cap styles of the end of a line.	String	Flat
*StrokeLineJoin*	Specifies how to join consecutive line segments in a figure	String	Flat
*StrokePentype*	Specifies the type of fill uses to fill lines.	String	SolidColor
*StrokeWidth*	Setting the width of a line	String	Miter
*Points+*	Setting the coordination of a polygon (X1; Y1 X2; Y2...)	String	33;34 31;31...
*Rectangle+*	Setting the boundary of a shape (X, Y, Width, Height)	String	2;2;10;10
*Border-Points*°	Setting the coordination of border	String	35;45 34;34...
*Border-Rectangle*°	Setting the boundary of border	String	3;3;10;20
*Border-StrokeColor*°	Setting the colour of border lines	String	0;; 0; 0
*BorderPaint*°	Set if the border should be shown.	Boolean	True
**Image**§	Stored binary data of the image	Binary	iVBORw0 KG...

Available (*) only in ToolML, (+) in VGraphML and MaskML, (°) in MaskML and (§) in VGraphML.

**Table 2:** List of attributes for textual information: the "Befund" element describes histological patterns of the PCa.

The root element:	Befund
**Attributes**	**Description**

*ID*	Identification number of PCa area
*ShowGleason*	Show the Gleason score in the pathology report
*Gleason1*	Primary Gleason pattern
*Gleason2*	Secondary Gleason pattern
*GleasonScore*	The Gleason Score
*Grading*	Gleason Grading
*Length, Width*	The size of the PCa area (Optional)
*SliceThick*	The thickness of the slice (Optional)

**Table 3:** List of attributes for textual information: the "Form" element.

The root element:	Form
**Attributes**	**Description**

*pT, pN, pM*	TNM-Classification
*Datum*	Date of the report
*Kernatypie*	Atypical changes in cell nucleus
*DiffMuster*	Grade of cell differentiation
*KlassifizierungAdeno*	Classification of adenocarcinoma
*GLS1*	Primary Gleason pattern
*GLS2*	Secondary Gleason pattern
*GLSTotal*	Total Gleason score
*GGrading*	Gleason grading
*HELPAP*	HELPAP specification
*L*	Lymph vessel invasion
*V*	Venous vessel invasion
*R*	Status of resection margin
*LK-B*	Number of lymph nodes with metastases
*LK-G*	Total number of extirpated lymph nodes

*ProstatLength, ProstatWidth* *Prostatvolumen*	Prostate diameters (Optional) Prostate volume

*BarcodeLIB*	Defining the barcode type (e.g. CODE128A)

### Analysis of the pathology report

The report of radical prostatectomy specimens (Figure 1) contains text and graphic data. Personal and pathologic information are textual. The anatomical scheme of the prostate gland is a template for the graphical documentation of histopathological findings. As previously published by Bettendorf et al. [6], the anatomical schema consists of both seminal vesicles and the prostate sectioned into eight defined slices. For each slice, a "slice factor" is attributed to estimate the tumour volume in relation to the total prostate volume. Symbols and icons are added to facilitate identification of the pathological findings. The report template was primarily designed for the documentation of PCa and High Grade Prostatic Intraepithelial Neoplasia (HGPIN), a presumable precursor for PCa [8,9]. PCa was graded according to Gleason [10] and Helpap [11] and staged using the TNM staging system (2002) [12].

The TNM-classification system [12] is a well-known pathological documentation system that codes tumour spread (T), lymph node invasion (N) and metastases (M). Parameters like residual (R), venous invasion (V) and lymph vessel invasion (L) provided additional clinical information about PCa. The Gleason score system is a standard applied to assess the histological pattern and spread of PCa [10]; Helpap is a method for the histological evaluation of cell differentiation and the existence of atypical prostatic cells [11].

### Document architecture of "cMDX"

cMDX document architecture was designed to meet the Open Packaging Convention (OPC) [13] (Figure 2 and additional file 1). Data were divided into template data and patient data (Figure 2 and 3). Each XML element in cMDX was declared by a corresponding class library. The element declaration was performed with the XML namespace "xmlns".

### Template data (TD)

The template data is stored in addition to the Background Image component (BGImage) in two different XML files: Mask.xml and Tools.xml. Tools.xml stores information about drawing tools applied to sketch pathological changes in scheme styles (e.g. freehand drawing) and provides parameters for the drawing tools. The xml container Tools.xml contains **DrawTool **elements. Each xml element **DrawTool **has two types of attributes:

(1) Explanatory attributes for pathological changes like *Description*, *Name *of a disease written in short form, *Image-Filename *for the symbolic representation of the disease, and *Text *for the full name of the disease.

(2) Attributes for graphical representation (Table 1), which are used for building a new graphic object to be displayed onscreen (Figure 4).

In addition, the optional **Properties **Element enables the interaction of the drawing tools with the application.

Mask.xml stores configuration data to define the appearance of the graphical template including a background *Image*, which is stored in BGImage, and vector shapes with clip function. These shapes represent the drawing surfaces onscreen. Three shapes were defined: (1) **Polygon **(2) **Rectangle **(3) **Ellipse**. Every shape element, whose tag name is the applied shape name, was stored inside the root element **Mask**. For instance, the element **Ellipse **represents a slice of the prostate. It has identification (e.g. *Id*, *Name*) and representation attributes (Table 1), which, for example, provide information about the *Percentage *volume of the slice in relation to the whole prostate volume (slice factor), the *PixelVolume *of the slice, and the *SizeOfSlice *for storing the real dimension of the prostate slice.

The component "BGImage" includes an Image representing the prostate and the seminal vesicles in schematic style and can be changed by the user. Various image formats (TIFF, BMP, WMF, PNG, JPEG, GIF and SVG) are hereby supported.

### Patient Data (PD)

Data acquisition from the prostatectomy specimen consists of two types of information: (1) morphometrical information about histological changes (e.g. PCa, HGPIN, positive surgical margin) with additional textual descriptions (Gleason Score, length of positive surgical margin), and (2) clinical and personal data in text form. Accordingly, two xml containers were designed: Form.xml and Map.xml. Map.xml captures morphometrical information about histological findings to reconstruct them in schematic styles. Diverse graphic objects were defined and stored in the root xml element **Map **(Figures 3 and 5). The representation of the spread pattern of PCa and HGPIN was based on vector graphics. The real dimension of a cancer area (*Length *and *Width*) as well as the slice thickness (*SliceThick*) can be optionally given for special scientific queries. In addition to the representation attributes, attributes like *Relation *and *RelationSlide *provide the topographical location of PCa foci in relation to each other. An XML Element called **Befund **was integrated into the CDATA-Section of a graphic object showing a PCa focus. **Befund **captures information about histological patterns of the corresponding PCa focus (Table 2).

Form.xml contains the element **Formular **with attributes containing personal data; pathological findings were categorised as textual information not directly related to the morphometrical data like prostate length, width and volume (ProstatLength, ProstatWidth, Prostatvolumen) (Table 3). Sensible personal data was encrypted with Rijndael 256-bit Cryptography [14] to avoid misuse by unauthorized users (Figure 5).

### Visualization Model

A Four-Layer model for visualization of graphical information was designed (Figure 6): (1) The Input Layer registers the cursor movement onscreen and the drawing tools selected by the user for data acquisition. (2) The Mask Layer defines the drawing surface. (3) The Draw Layer represents the drawing elements resulting from the Layers 1 and 2. (4) The Background Layer contains schematic diagrams with graphical descriptions as images.

### Clinical evaluation of cMDX for estimating tumour volume and multifocal PCa

Two-hundred and fifty-five patient records were chosen randomly for a retrospective study, in which their reports of radical prostatectomy specimens were digitalized and converted into cMDX reports. We analysed PCa foci within the prostate boundary. Therefore, a C#-based tool was implemented to analyse cMDX documents. Results including mapping, tumour volume, multifocality and incidence of PCa in each slice were exported as CSV files and analysed with PASW^© ^Statistics version 18 (SPSS Inc., Chicago, United States). At the same time, an experienced pathologist estimated tumour volume and multifocality of PCa by eyeball judgment alone. These results were then compared with the results of the analysis tool.

### Cancer volume estimate

In the pathology institute the prostate volume (*Prostatvolumen*) was calculated after formalin fixation by weighing the prostate specimen without the seminal vesicles. For the purpose of our study, the prostate weight in grams is considered roughly equivalent to its volume in cubic centimeters (cm^3^); the tumour/entire gland ratio is then used to calculate the volume of the tumour in cm^3^. In addition, the diameters of prostate specimens were recorded (*ProstatLength*, *ProstatWidth*). A correction factor for tissue shrinkage after formalin fixation was not applied. The computational tumour volume estimate was performed on the basis of the Bettendorf's scheme [6]: The tumour area in each slice in the diagram is estimated by counting the pixels which contain PCa. The cancer area is divided by the slice area (pixel) and then multiplied with the slice factor to calculate the relative cancer volume. Finally, the total relative cancer volume is multiplied with the prostate volume to estimate the cancer volume in cm^3^.

## Results

### cMDX Editor

**Figure 7:**
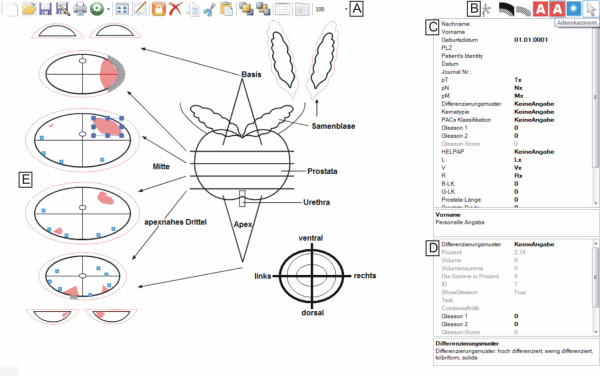
**The Graphical User Interface of cMDX Editor**. A: tool bar of the cMDX Editor (new template, open, save, save as...); B: drawing tools for documenting histological changes; C: input field for personal and clinical data; D: window for viewing the histological properties of the selected PCa area. E: the drawing surface.

**Figure 8:**
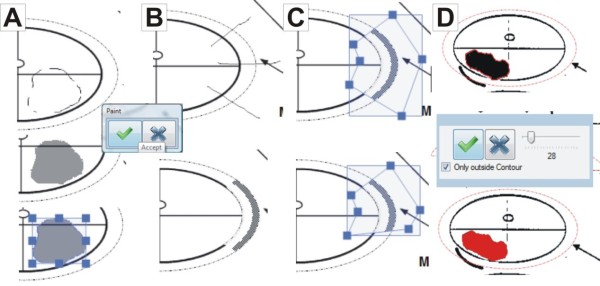
**How to draw A: a PCa area, B: capsular invasion, and C: modify the extent of capsular invasion. D: Magic Wand Tool**. A: The pathologist sketches the boundary of the PCa area. After clicking "accept", the boundary is completed automatically. B: The user sketches lines to describe a capsule invasion. C: The user can modify the extent of the capsule invasion by selecting and moving the blue squares shown above. D: The "magic wand tool" allows selecting areas marked on the background image by selecting "only outside contour," the tool marks only the outside contour of the focus; otherwise, the inside contours are included.

The cMDX Editor assists users in generating cMDX documents. Figure 7 illustrates the user interface of this application. The graphical presentation of the schematic diagram was processed according to the Four-Layer model described above (Figure 6). Each pathological finding was assigned to a drawing tool element (Table 4 and Figure 8). For example, the user may create a polygenic shape to present the boundary of a PCa focus. The magic wand tool based on the 8-flood fill algorithm was provided to indicate PCa foci marked on the background image. The user can change the background image and the properties of the defined drawing surfaces. An overview of the pathological parameters and estimation of the tumour volume is included in the electronic report (Additional file 2).

**Table 4:** List of drawing tools.

Symbol	Description	DrawTool (*Type*)
	High grade Prostatic Intraepithelial Neoplasia	ImageWithoutSizing
	Adenocarcinoma	Polygon
	Adenocarcinoma	FloodFill
	Capsular invasion	Clip (*TypeOfClip*: Polygon)
	Extracapsular extension	Clip (*TypeOfClip*: Polygon)
	positive surgical margin	ImageWithoutSizing

### Clinical Evaluation of cMDX

The cMDX documentation system was successfully connected to the local electronic Hospital Information System (HIS). The cMDX Editor can generate reports in Portable Document File (PDF) format to be imported into the corresponding electronic patient record. The complete electronic documentation took on average about eleven minutes (mean: 11 ± 2 min STD). The electronic documents were preferred by pathologists and urologists because of the clear and unambiguous presentation of the pathological findings.

**Figure 9:**
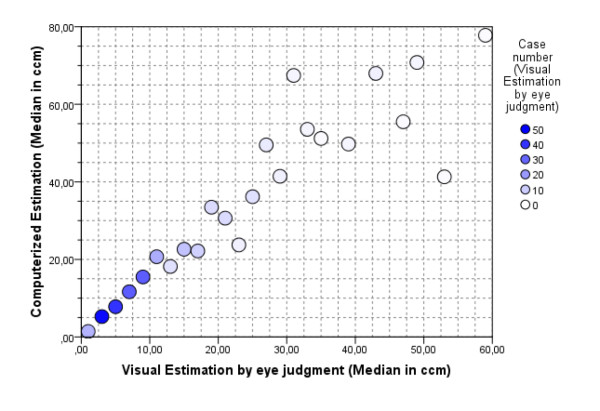
**Scatter plot comparing computerised with visual estimation of tumour volume in 255 prostatectomy specimens**. Increasing discrepancy in tumour volume estimation between visual and computerised estimations was found with increasing tumour volume. The eyeball judgment resulted in lower PCa volume estimation in most cases.

**Figure 10:**
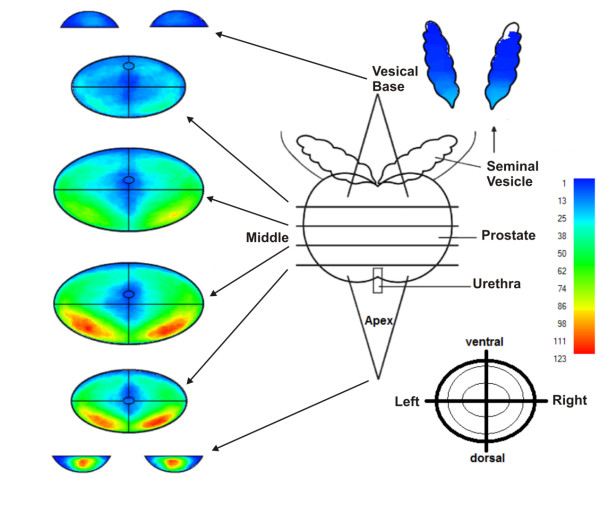
**The cumulative diagram of 1615 PCa foci in 255 prostatectomy specimens**. The cumulative diagram of 1615 PCa foci shows that prostate cancers are mostly localized in the peripheral zone, and that toward the base PCa foci seem to diverge laterally. PCa seems to be frequently located in the dorsal half of the peripheral zone of the prostate. The frequency of tumour foci in the various locations are coded by colours, blue = low, red = high frequency.

Two-hundred and fifty-five reports of radical prostatectomy specimens were digitalized using the cMDX Editor. The computerized estimation of tumour volume showed that the mean tumour volume was 17 ± 16.4 cm^3^. In comparison, the mean tumour volume estimated by eyeball judgment was 10 ± 10.3 cm^3^. Thus there is a significant dissimilarity in tumour volumes between the two different estimation methods (mean difference = 6.6 ± 8.7 cm^3^, T = 12.02, DF = 254, p < 0.0001) (Figure 9). In our patient population, 75% of PCa were staged as pT2c or pT3a. Statistically, there is a highly significant correlation between T-staging and tumour volume (spearman rho = 0.513, p < 0.01). The cumulative diagram of 1615 PCa foci revealed that prostate cancers are mostly localized in the peripheral zone (median: 83.5%, mean: 73 ± 25%) and toward the base PCa foci seem to diverge laterally (Figure 10 and Table 5). 52.5% of PCa were multifocal. The analysis tool could detect 90.3% of PCa identified as multifocal by eyeball judgement (Table 6). This analysis of 255 cMDX reports had taken 20 ± 2 seconds on a notebook with Intel^® ^T5600 Core™ Duo 1.83, RAM: 4 GB and Nvidia^® ^Geforce^® ^Go 7300 128 MB.

**Table 5:** The spread pattern of PCa in PZ.

Section Level of Prostate	PC Localized in PZ
Base	36%
Middle 1	80%
Middle 2	87%
Apex	89%

**Median**	83.5%

**Mean**	73% +/- 25

**Table 6:** Multifocality of PCa & detection rate of multifocal PCa.

		Control		
	**PCa**	**Unifocal**	**Multifocal**	**Total (%)**
**Analysis Tool**	Unifocal	121	13	134 (52.5)
	Multifocal	0	121	121 (47.5)
Total (%)		121 (47.5)	134 (52.5)	255 (100)

Sensitivity of Analysis Tool for detecting multifocal PCa: 90.3%

## Discussion

cMDX is a data acquisition model for graphical and textual information. Sensible data were encrypted with 256-bit cryptographic algorithms to enable data analysis compliant with data privacy regulations. The visualization model based on the Four-Layer Model combined a graphical overview of tumour growth in a schematic style with the related textual information. In this manner, the electronic documentation of histopathological information usually given by reports in paper form is realised.

XML enables to extract clinical data and graphical schemes, and to transmit them into different formats like binary graphics (PNG, JPEG) vector graphics (SVG), Portable Document Files (PDF), text files (CVS), and Client Report Definition (rdlc) [15]. Therefore, cMDX can be converted into a data format supported by the HIS. The file architecture is compatible with open package conventions and therefore standard documents like HL7 CDA can be integrated into cMDX [16].

The software prototype of the cMDX Editor can generate and modify pathology reports according to the clinical requirements of pathologists and urologists. The integration of reports of radical prostatectomy specimens into the HIS improves clinical utilities and acceptance by the clinicians.

The application provided an estimate of the tumour volume, which may be an important prognostic indicator for predicting prostate cancer recurrence following surgery [17]. In the literature several methods to determine tumour volume of PCa can be found. These include visual estimation, computer planimetry with computer-assisted image analysis. Computer planimetry of the full set of slides of a serially sectioned prostate is believed to be the best technique for an accurate measurement of tumour volumes; however, this technique is expensive and time consuming and is therefore not applied in routine clinical practice [18]. The estimation of tumour volume by eyeball judgment is simple and less time consuming, and cheap but shows less precision of measurement [18], it is performed directly under the microscope or after the transformation of tumour areas into the reconstruction map of the prostate in a schematic style [6]. Our results show that the estimation by eyeball judgment alone resulted in lower tumour volumes as compared with cMDX estimation. The computerised estimation of tumour volume with the cMDX Editor seems to be more accurate than the estimation by eyeball judgment. We anticipate an increase in accuracy of eyeball judgement with a grid method getting close to the accuracy of the computational volume estimation. Nevertheless further investigations are needed here. However, these estimation methods are not accurate in comparison to the computer planimetry because they exclude the real dimensions of the prostate and the PCa foci.

The anatomical representation of the prostate in schematic style facilitates collecting and analysing the spread pattern of PCa in the prostate. For instance, such information can be helpful to assess the biopsy strategy in order to increase the detection rate of PCa [19-21]. Our approach enables to analyse cMDX reports and export the results into programs like SPSS^© ^or Excel^©^. Our results confirm the consensus that the major tumour mass of PCa is located in the peripheral zone (PZ) [22]. According to Chen et al., 74% of PCa foci were localized in the PZ and toward the base, diverged laterally [23]. We found a significant correlation between tumour volume and pathological stage of the specimens, thus confirming the findings of Nelson et al. [24]. The current analysis tool can detect multifocal PCa with a sensitivity of 90.3%. The multifocality rate of PCa was 52.5%, which is consistent with prior series that reported multifocality rates of 50 to 87% [25]. The accuracy to differentiate multifocal from unifocal PCa depends on the applied algorithm. The transformation of the tumour area into a rectangular shape increases the size of the tumour area and increases the probability of one focus to overlap with another adjacent focus in the vicinity, this way reducing the detection rate of multifocal PCa.

A limitation of our documentation model is the documentation time necessary, cMDX failed to shorten the documentation time. cMDX is still developing and we will focus on this drawback in future versions.

An electronic documentation standard for pathologic findings of prostate specimens has not yet been introduced. We conducted a Pubmed search using the key words "electronic documentation prostate", "electronic report prostate" and "electronic documentation prostatectomy." Only one software program similar to the cMDX Editor could be found: PixelProstate (freeware), developed by Nickels [26], mainly focuses on the measurement of tumour volume. PixelProstate has an internal database to store clinical data and provides a simplified 3 D illustration of the prostate and PCa foci. To document the patterns of the tumour spread, the PCa foci are drawn using multiple circles. The documentation of histological patterns of each PCa focus is not supported. In contrast, the cMDX Editor enables to convert a pathologic report into a portable file format and to draw PCa foci with the freehand drawing tool. In addition, capsular invasion, extracapsular extension, positive surgical margins, and histological patterns of a PCa focus can be documented, but a 3 D illustration model similar to that of PixelProstate is not available. cMDX is a free open source document architecture. The applications based on cMDX (cMDX Editor and Analysis tool) are still prototypes (Beta-Version). We plan to develop two versions of these applications (commercial and non-commercial) and make them available as soon as possible.

cMDX offers an extensible document architecture by adding a new drawing tool and by providing additional class libraries. If needed, additional pathological parameters or characteristics can be added. For instance, recording the Gleason score of a PCa focus is feasible by adding the class library "eMocS.Definition.Befund" from the corresponding XML part of cMDX. Furthermore, this feature could extend the spectrum of the clinical applications of cMDX to various medical applications, e.g. for protocols for cardiac catheterisation or extension estimates of burn injuries in emergency surgery.

## Conclusions

cMDX can be applied to store clinical data in schematic styles for reporting and analysing pathological parameters in radical prostatectomy specimens. It facilitates to evaluate routine data for scientific purposes.

## Competing interests

The authors declare that they have no competing interests.

## Authors' contributions

OE designed the cMDX architecture, programmed the supplementary tools and performed the statistical analysis. AS and OB designed the paper template for the pathologic report. AS and OE designed the template for the pathology report in electronic form. AS, EE, JN, MA, OB, OE, RH and TK acquired the data and reviewed the implementation of the software prototype. AS, OE and RH reviewed the results of the analysis. OE drafted the manuscript. AS and MD reviewed the manuscript. All authors read and approved the final manuscript.

## Pre-publication history

The pre-publication history for this paper can be accessed here:

http://www.biomedcentral.com/1472-6947/10/71/prepub

## Supplementary Material

Additional file 1 **An example cMDX file "Example.cMDX"**. Please open the file using a zip programme to view the document architecture. Click here for file

Additional file 2 **An example of a report in electronic form "Example_ElectronicForm.pdf"**. The paper version of this report is depicted in Figure 1. Click here for file
