# Supplementary material for: Clinical map document based on XML (cMDX): document architecture with mapping feature for reporting and analysing prostate cancer in radical prostatectomy specimens
Source: BMC Med Inform Decis Mak. 2010 Nov 15;10:71. doi: 10.1186/1472-6947-10-71 (PMC2995775; doi:10.1186/1472-6947-10-71)
Supplement: Additional file 2 — An example of a report in electronic form "Example_ElectronicForm.pdf". The paper version of this report is depicted in Figure 1. [file 1472-6947-10-71-S2.PDF]

Prostatazentrum am UKM

UKM Universitätsklinikum Münster

9999/99

Morphometrical Examination of prostate cancer for

Mustermann, Manfred

99.99.9999

J. Nr. 9999/99

M.D. Peter (Pathologist)

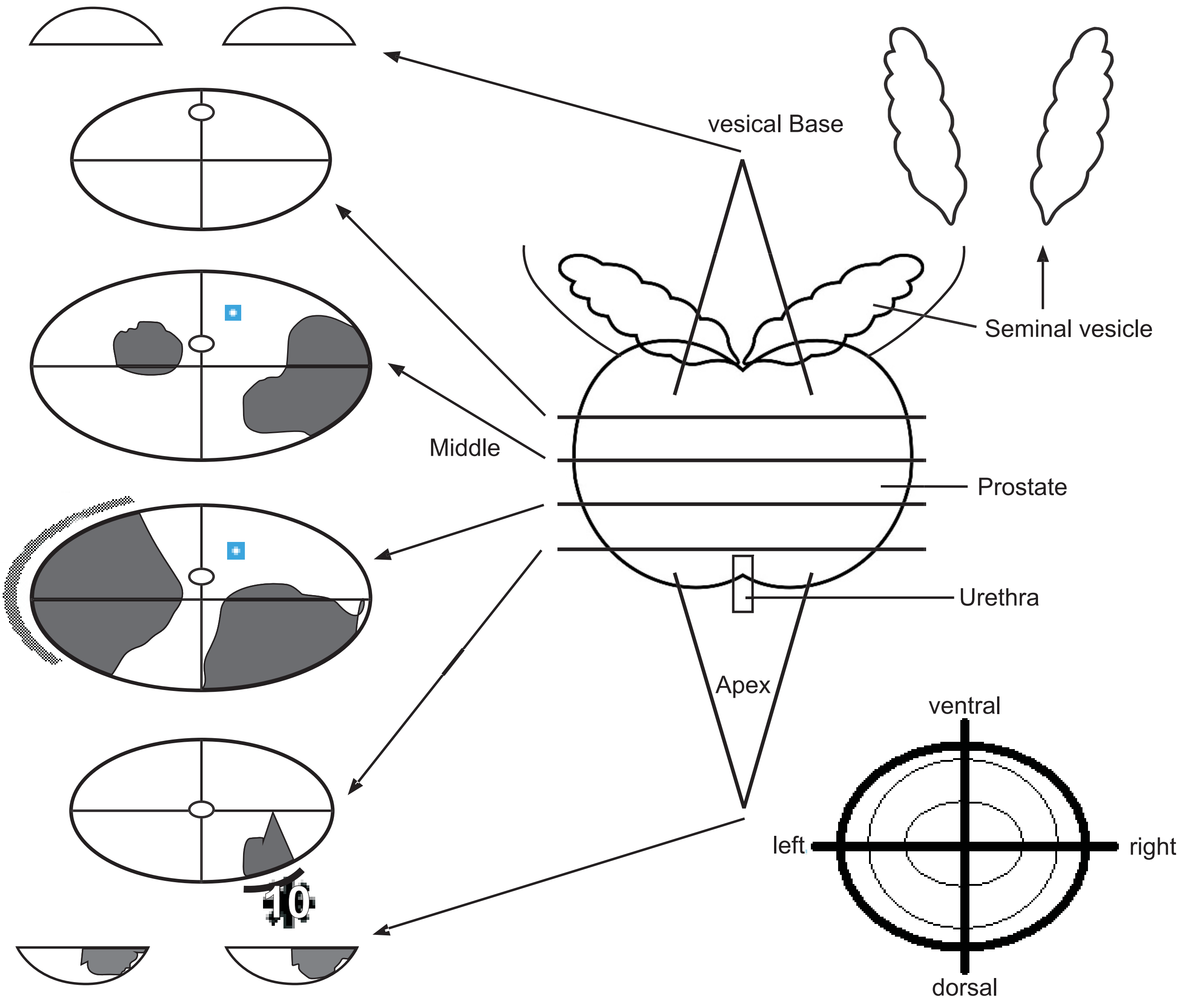

	Prostatic Intraepithelial Neoplasia (high grade PIN)	Yes		
	Adenocarcinoma	Yes	15 %	7 ccm
	Capsular Invasion	Yes		
	Extraprostatic tumor extension	Yes		
	positive surgical margin	Yes		

The depth of positive surgical margin in mm.

Classification pT3a pN0(0/10) L0 V0 Local-R1

Gleason-Score: **3 + 3 = 6**

Helpap grading: IIa

Prostate volume: 50 ccm

Comments:
